# R0 resection and reconstruction for a large, rapidly progressive chest wall sarcoma

**DOI:** 10.1186/s13019-018-0812-8

**Published:** 2018-12-17

**Authors:** Robert L. Kress, Shraddha M. Dalwadi, Adel D. Irani

**Affiliations:** 1 Department of Surgery, UT Health at McGovern Medical School, 6431 Fannin St, Houston, TX 77030 USA; 2 Department of Cardiothoracic And Vascular Surgery, UT Health at McGovern Medical School, Houston, USA

**Keywords:** Soft tissue sarcoma, Leiomyosarcoma, Chest wall sarcoma, Wide local excision, Chest wall reconstruction

## Abstract

**Background:**

Chest wall sarcomas are a rare group of soft tissue malignancies with variable presentations. Here we describe the definitive management of a large, rapidly progressing chest wall sarcoma arising from the pectoralis major muscle.

**Case report:**

An obese 42-year-old African American male with multiple medical comorbidities presented with new onset right-sided chest pain and a palpable right chest mass. Initial CT chest demonstrated a 9x9x9cm necrotic mass arising from the pectoralis major. CT-guided core biopsy was positive for high-grade spindle cell neoplasm (positive for smooth muscle actin, desmin, S100, and CD31; negative for CD34, PAX8, and beta-catenin). Staging imaging 2 months later demonstrated growth of the mass to 21.4 × 17.8 × 13.7 cm. The patient underwent neoadjuvant chemoradiation with surveillance CT imaging demonstrating a stable tumor. Then he underwent wide local excision of the mass followed by delayed local myocutaneous flap reconstruction and skin grafting. Final pathology was R0 resection, 38x20x18 cm tumor with 70% gross necrosis. Microscopic examination confirmed high-grade sarcoma with smooth muscle differentiation. Final pathologic staging was Stage III G3 pT2bNxMx.

**Conclusions:**

This patient presented with a rare, rapidly enlarging high-grade leiomyosarcoma of the chest wall without metastases or violation of the thorax. We describe the definitive management including a multidisciplinary team to manage a complex and rapidly progressive sarcoma of the chest wall.

## Introduction

Primary chest wall sarcoma is a rare disease process, comprising less than 10% of the 8000 sarcoma cases diagnosed annually in the United States [1, 2]. Most studies investigating this malignancy are retrospective small series or case reports with a variety of treatment approaches. The prognosis and clinical outcome of chest wall sarcomas are thought to be similar to extremity sarcomas [3]. For this reason, chest wall sarcomas are often treated similarly to extremity sarcomas with wide local resection +/− neo/adjuvant chemoradiation. Herein, we describe the definitive management of a rapidly enlarging, extensive chest wall sarcoma originating from the pectoralis major muscle.

## Case report

An obese 42-year-old African American male with diabetes mellitus, hypertension, heart failure with reduced ejection fraction, coronary arterial disease, and atrial fibrillation presented with new onset right-sided chest pain and a palpable right chest mass. Ultrasound showed an anterior right chest, well-demarcated 7.5 × 6.5 × 4.8 cm, intramuscular mass, 0.6 cm deep to the skin. CT demonstrated a 9x9x9cm necrotic mass arising from the pectoralis major. CT-guided core biopsy was positive for high-grade spindle cell neoplasm (positive for smooth muscle actin, desmin, S100, and CD31; negative for CD34, PAX8, and beta-catenin) and verified by two independent pathologists.

Unfortunately, the patient was lost to follow up after referral to Oncology and Thoracic Surgery. He presented to oncology clinic with progressive symptoms including a rapidly enlarging chest mass, increasing pain, and new onset chest wall numbness. A repeat CT showed that the mass increased in size to 21.4 × 17.8 × 13.7 cm without evidence of metastatic disease over course of two months. MRI was consistent with 23 cm mass within the right pectoralis major without vascular or bony invasion. The patient chose to undergo neoadjuvant chemotherapy given his multiple comorbidities. He was hesitant to pursue resection and elected to attempt to shrink the tumor before resection.

Patient underwent neoadjuvant chemotherapy with gemcitabine and docetaxel for 2 cycles followed by radiation therapy (50Gy over 2 months to tumor bed + 3 cm margins). Restaging CT showed a stable tumor at 23 cm without any evidence of local or distant metastases.

**Fig. 1:**
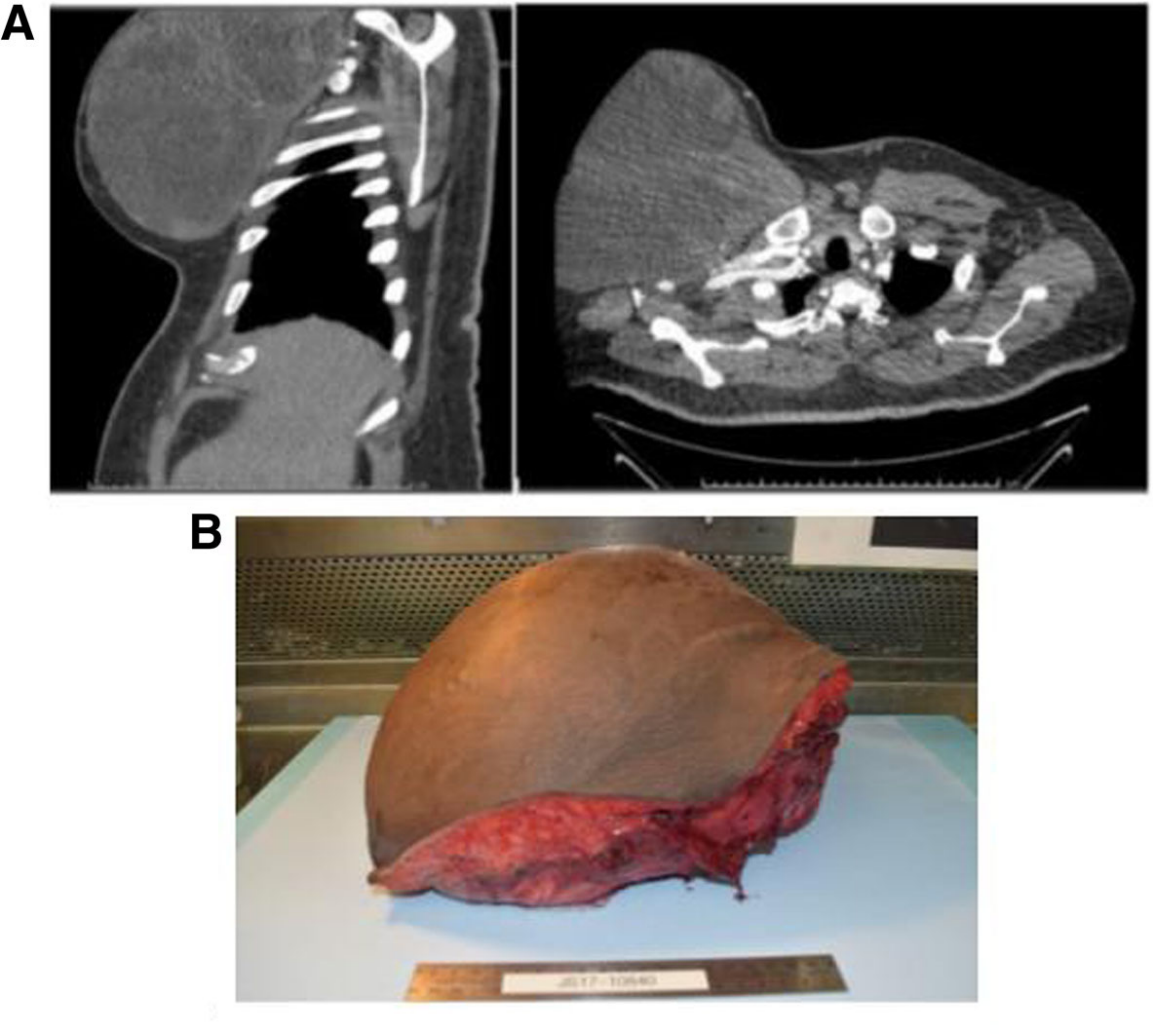
**a** Preoperative CT Chest **b** En bloc resection chest wall sarcoma

**Fig. 2:**
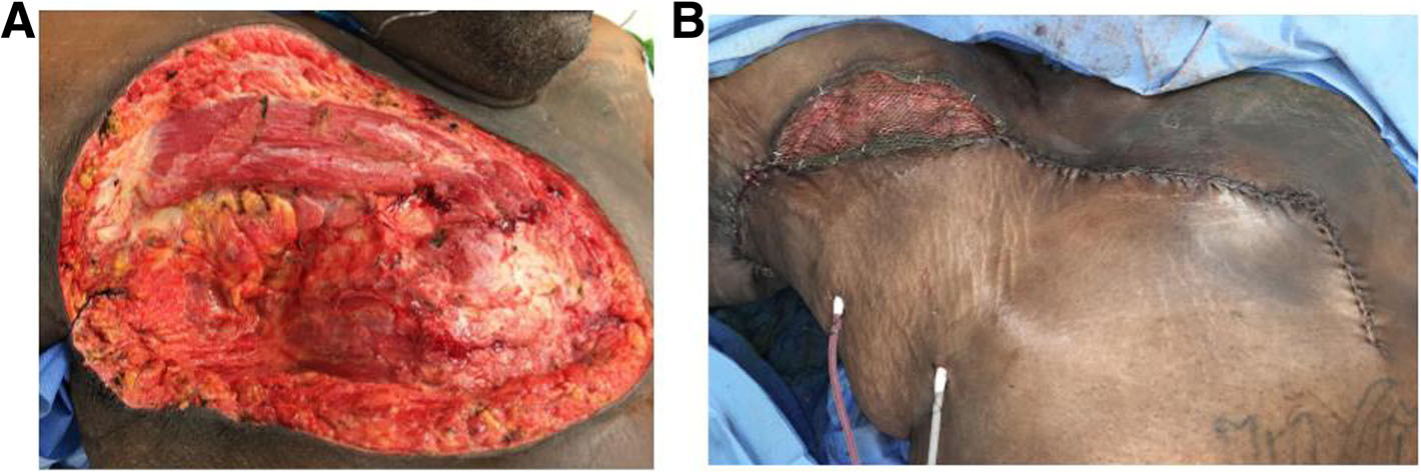
Large Chest wall defect s/p wide local excision. **a** 1,000cm^2^ defect with exposed pectoralis, intercostal muscles and bone. **b** defect s/p myocutaneous local flap and skin grafting

The patient returned to Thoracic surgery clinic to discuss options for resection and reconstruction. He then underwent wide local surgical resection for definitive therapy. Tumor was removed en bloc with resection to the intercostal fascia including pectoralis major and minor (Fig. 1). There did not appear to be any violation of the intercostal investing fascia and no entry into the thoracic cavity was made. Negative pressure dressing was used until post-operative day 7 when Plastic Surgery performed advancement flap coverage and skin grafting to the > 1,000cm^2^ defect (Fig. 2a, b).

**Fig. 3:**
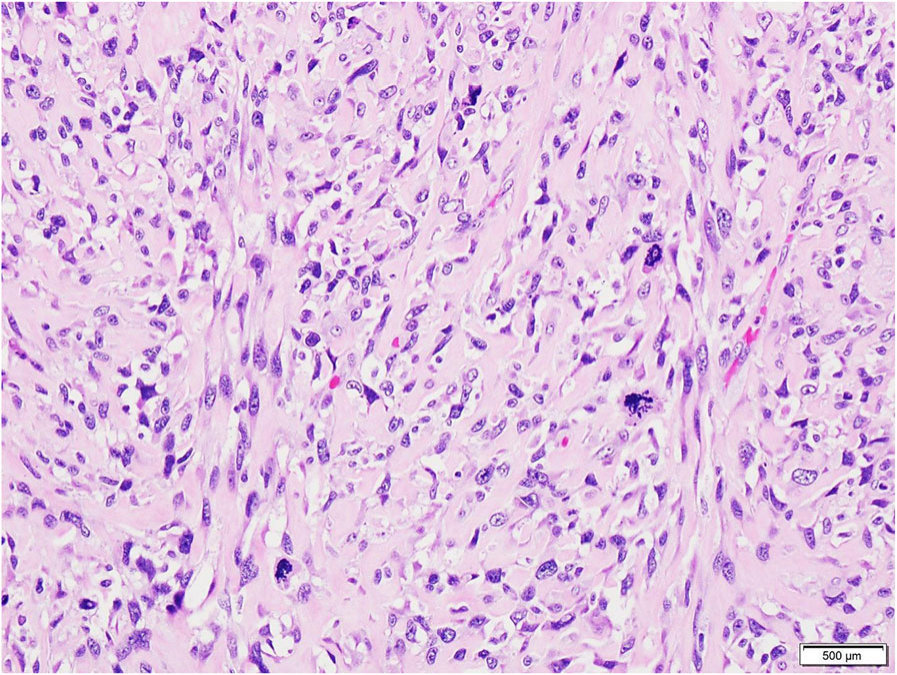
Representative H&E slide demonstrating smooth muscle differentiation of the high-grade sarcoma

Final pathology demonstrated 38x20x18 cm tumor with 70% gross necrosis and R0 resection. Microscopic examination confirmed high-grade sarcoma with smooth muscle differentiation (Fig. 3). Final pathologic staging based on AJCC 7th edition was Stage III G3 pT2bNxMx.

## Discussion

Soft tissue sarcomas (STS) are a group of tumors with mesenchymal origins including bone, nerve, muscle, and adventitia. They are a rare disease which makes definitive prospective study difficult. To date, the largest study groups of STS have been retrospective with given limitations. Current recommendations for STS of the chest wall include wide local or radical surgical resection to achieve R0 margins as this is associated with a lower local recurrence rate compared to R1 or R2 margins [4–6]. Neoadjuvant chemotherapy is not recommended in cases of resectable STS tumors. Our patient was counseled that any delay in resection could result in further tumor local invasion or metastases [7, 8].

The rate of radical resection including need for chest wall reconstruction is high in STS of the chest wall. The most common area of thoracic wall resection is anterior, posterior, followed by flank with most common type of reconstruction being mesh [5, 9]. Our patient underwent delayed reconstruction, within 7 days, as there was no thoracic wall defect necessitating immediate reconstruction. He had a large soft tissue defect that was temporized and covered with local myocutaneous advancement flaps before discharge at the index admission.

Overall survival for STS of chest wall have been described from 33 to 88.5% at 5 years [9–13]. Evidence for adjuvant chemoradiation was suggested by Burt et al. which demonstrated a reduction in disease-free survival by 89% when added to surgery alone. However, overall survival trended toward a reduction in mortality but was not significant [14].

Here we demonstrate definitive treatment of a rapidly enlarging leiomyosarcoma arising from the pectoralis major muscle. This patient underwent neoadjuvant chemoradiation with stable tumor size and extensive tumor necrosis on final pathology. His case is unique with large size at presentation, rapid growth without penetration into the thoracic fascia or cavity, and leiomyosarcoma on final path. To our knowledge this is the first case presented of such a large, high grade leiomyosarcoma. Previous retrospective studies and case series describe STS of the chest wall ranging from 3 to 32 cm with leiomyosarcomas encompassing 2–10% of reported STS [5, 9, 11, 14]. This patient had a tumor removed more than double the size of the reported range for leiomyosarcomas without violation into the thoracic cavity or metastases at the time of surgery.

Recommended follow up includes every 4–6 month physical exam with CT chest every 6–12 months for the first 3 years then yearly thereafter. These recommendations are extrapolated from data demonstrating 89% of recurrences occurring within 3 years of index operation [4]. Predictors of recurrence have been described as high grade tumor on pathology, size > 5 cm, high uptake on PET/CT [4, 15]. This patient was followed by a multidisciplinary team including Oncology, Plastic Surgery, Thoracic Surgery for his continued surveillance as recommended for STS of the chest wall [16]. Unfortunately, on repeat CT Chest he developed diffuse pulmonary metastases in the 6 months following surgery. He was referred back to oncology for evaluation for chemotherapeutic treatment.

## Conclusions

Here we describe the definitive management of an extensive, progressive soft tissue sarcoma of the chest wall. STS should be managed by a multidisciplinary team of surgeons, medical oncologists, and radiation oncologists. This patient presented with a large, rare tumor of the chest wall without metastases or intrathoracic extension of tumor. He chose to undergo neoadjuvant chemoradiation with tumor progression. He then underwent R0 resection of the sarcoma with subsequent local myocutaneous reconstruction. Large tumors of the chest wall can be managed aggressively with wide local excision or radical resection, and delayed or immediate reconstruction.

## Abbreviations

(STS): Soft tissue sarcomas
